# Long noncoding RNA LINC00115 promotes breast cancer metastasis by inhibiting miR‐7

**DOI:** 10.1002/2211-5463.12842

**Published:** 2020-06-11

**Authors:** Chunlei Yuan, Xuliang Luo, Sijia Duan, Liangyun Guo

**Affiliations:** ^1^ Department of Breast the Second Affiliated Hospital of Nanchang University China; ^2^ Department of Ultrasound the Second Affiliated Hospital of Nanchang University China

**Keywords:** LINC00115, metastasis, miR‐7, triple‐negative breast cancer

## Abstract

Breast cancer is the second leading cause of cancer‐related deaths in women. The long noncoding RNA LINC00115 has been reported to be involved in the poor outcome of patients with breast cancer, but the biological function and underlying mechanism remain unclear. Here, we report that LINC00115 expression is increased in triple‐negative breast cancer tissue compared with matched normal tissue, and LINC00115 knockdown suppresses breast cancer cell migration and invasion. Furthermore, we show that LINC00115 directly targets miR‐7 and inhibits its expression. LINC00115 also reduces the expression of KLF4, which is a direct target of miR‐7 and is involved in breast cancer metastasis. Together, our findings suggest that LINC00115 promotes breast cancer metastasis through modulating the expression of miR‐7 and KLF4.

## Graphical abstract

LINC00115 expression is increased in breast cancer tissue and promotes tumor cell migration and invasion by inhibiting miR‐7 expression. This miRNA has been identified as a tumor suppressor in breast cancer, and one of its direct targets, KLF4, is an oncogene in breast cancer.

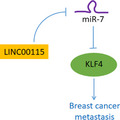

**Abbreviations**
lncRNA: long noncoding RNA
qRT–PCR: quantitative reverse transcription polymerase chain
ROC: receiver operating characteristic
TNBC: triple‐negative breast cancer

Breast cancer is the most common malignancy and the second leading cause of cancer‐related deaths in women. It was established that 266 120 women were diagnosed with invasive breast cancer and 40 920 women died from breast cancer in the United States in 2018 [1]. Triple‐negative breast cancer (TNBC) is a cancer without estrogen receptors, progesterone receptors, and excess HER2 protein, and includes three common features: (a) more aggressive and poorer prognosis than other types of breast cancer; (b) be higher grade than other types of breast cancer; and (c) usually be like ‘basal‐like’ [2]. Approximately 10–20% of breast cancers are TNBCs [2]. According to the American Cancer Society report, the 5‐year survival rate of patients with stage IV (metastatic) breast cancer is 22%. Poly‐adenosine diphosphate‐ribose polymerase inhibitors are effective drugs to treat TNBCs with BRCA1 or BRCA2 mutation, but just about 30% of TNBCs are with BRCA mutation [3]. Therefore, exploring novel therapeutic target for TNBC is still urgent.

Long noncoding RNAs (lncRNAs) are a large class of single‐stranded RNAs with more than 200 nucleotides in length and without ability to encode proteins [4]. In the past decades, lncRNAs as tumor suppressor or oncogene have been confirmed to affect the progression of cancer [5]. In breast cancer, for example, lnc‐BM is upregulated in the tumor tissue and its overexpression is positively correlated with the poor patient survival, and lnc‐BM promotes the breast cancer cells to spread into the brain [6]. An increasing number of lncRNAs have also been reported to participate in breast cancer development, such as MALAT1 [7], AK023948 [8], and GAS5 [9]. Recently, Xu *et al*. [10] integrated the GEO data and TCGA data, as well as their own RNA‐sequencing data, and revealed hundreds of oncogenic lncRNAs in breast cancer, among which LINC00115 is involved in the survival time of patients with breast cancer and do not respond to estrogen. Otherwise, the previous study has also reported that LINC00115 may interact with miR‐7 to regulate lung cancer progression [11]. However, the role of underlying mechanisms of LINC00115 in TNBC remains to be explored.

In the present study, we found that LINC00115 expression is increased in TNBC tissue compared with matched normal tissue, and LINC00115 knockdown inhibits the migration and invasion of breast cancer cells. Furthermore, we demonstrated that LINC00115 directly represses the expression of miR‐7, which has been confirmed to suppress breast cancer metastasis.

## Results

### LINC00115 expression is frequently increased in TNBC and is associated with the poor overall survival

**Fig. 1:**
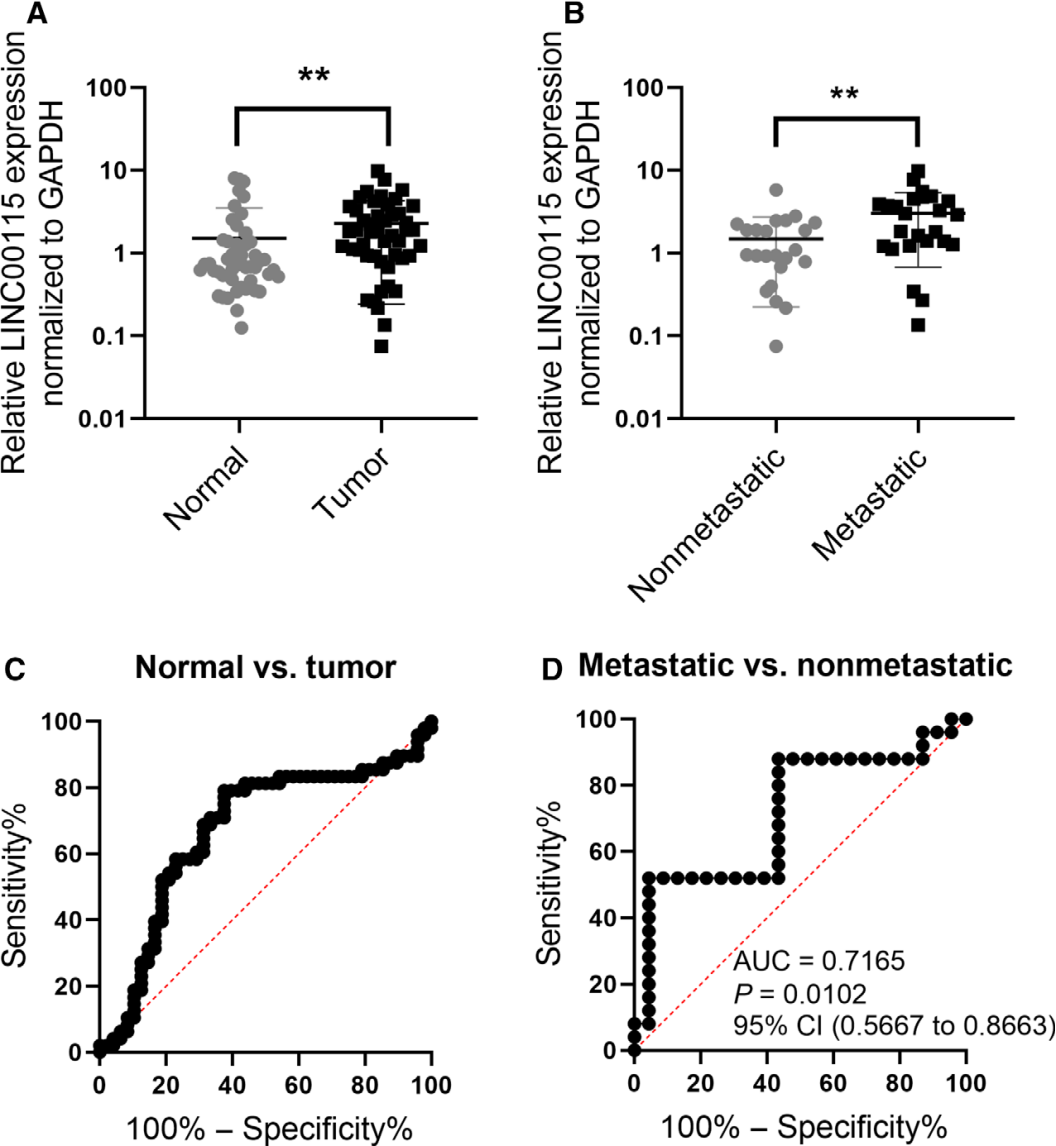
LINC00115 expression is increased in TNBC tissues and associated with lymph node metastasis. (A) Scatter plots show the expression of LINC00115 in 48 matched pairs of TNBC and normal tissues. (B) Scatter plots show the expression of LINC00115 in 25 lymph node‐metastatic TNBC tissues and 23 nonmetastatic TNBC tissues. qRT–PCR was used to detect the LINC00115, and GAPDH mRNA served as an internal control. The Mann–Whitney–Wilcoxon test was used. (C) ROC curve analysis for the expression signature of LINC00115 in distinguishing 48 TNBC tissues from the matched normal tissues. (D) ROC curve analysis for the expression signature of LINC00115 in distinguishing 25 lymph node‐metastatic TNBC tissues from 23 nonmetastatic TNBC tissues. **P < 0.01.

To determine the expression of LINC00115 in TNBC tissues, we collected 48 pairs of tumor tissues and matched normal tissues from 48 patients with TNBC, 25 among whom presented with lymph node metastasis. We detected the expression of LINC00115 in the tissues using quantitative reverse transcription polymerase chain (qRT–PCR) experiments, and the results showed that LINC00115 expression was significantly increased in TNBC tissues compared with matched normal tissues (Fig. 1A), and also elevated in lymph node‐metastatic TNBC tissues than in nonmetastatic TNBC tissues (Fig. 1B). These suggest that LINC00115 upregulation is associated with metastatic TNBC.

We further determined whether the expression signature of LINC00115 can distinguish lymph node‐metastatic TNBC from those nonmetastatic TNBC. Receiver operating characteristic (ROC) curve analyses showed that LINC00115 expression signature has an AUC of 0.6732, with sensitivity of 79.17% and specificity of 62.5%, in distinguishing the TNBC tissues from the matched normal tissues (Fig. 1C), and also has an AUC of 0.7165, with sensitivity of 52% and specificity of 95.65%, in distinguishing the lymph node‐metastatic TNBC tissues from the nonmetastatic TNBC tissues (Fig. 1D). These suggest that the expression signature of LINC00115 may serve as a potential biomarker for the prognosis of patients with TNBC.

### LINC00115 promotes breast cancer cell migration and invasion *in vitro*

**Fig. 2:**
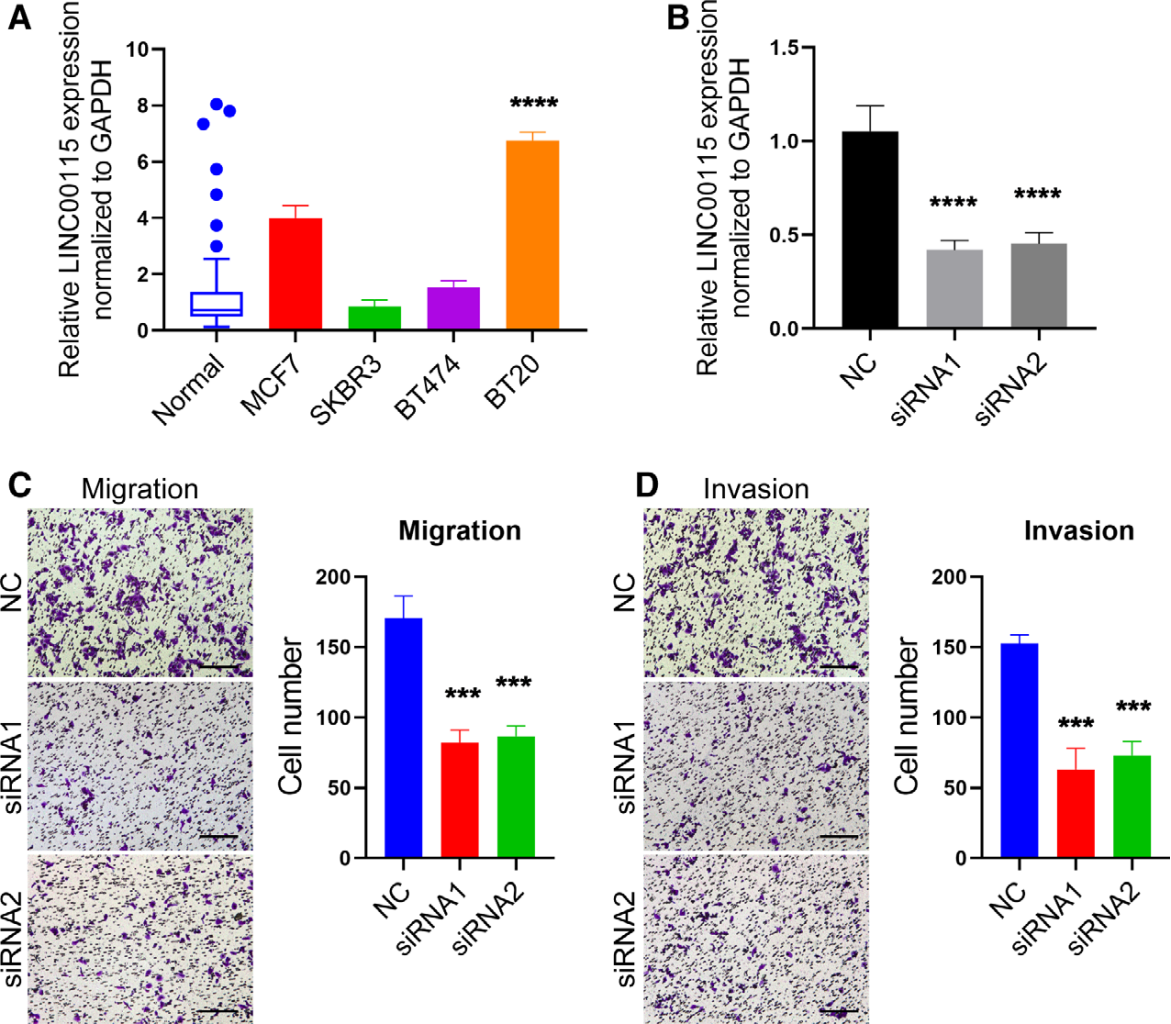
LINC00115 promotes breast cancer cell migration and invasion *in vitro*. (A) Box and whiskers show LINC00115 expression in 48 normal breast tissues; whiskers: Tukey. Bars show LINC00115 expression in four breast cancer cell lines. (B) Bars show LINC00115 expression in BT20 cells which were transfected with two specific siRNAs of LINC00115. qRT–PCR was used to detect the expression of LINC00115, and GAPDH served as an internal control. (C) Representative micrographs (×100) show the migration of BT20 cells, which were transfected with siRNA1 or siRNA2, respectively. (D) Representative micrographs (×100) show the invasion of BT20 cells, which were transfected with siRNA1 or siRNA2, respectively. Scale bars represent 25 μm. These experiments were repeated three times. Student’s *t*‐test and the one‐way ANOVA, followed by Tukey’s *post hoc* test, were used. All bars represent data with SD. ***P < 0.001; ****P < 0.0001.

To determine the biological functions of LINC00115 in breast cancer cell, we selected four breast cancer cell lines (MCF7, SKBR3, BT474, and BT20). We then chose BT20 cells to investigate the biological functions because it is a TNBC cell line with an invasive characteristic [12], and has the highest expression of LINC00115 compared with other cell lines (Fig. 2A). Next, we conducted the loss of function using the specific siRNAs for LINC00115 and confirmed that these siRNAs could markedly decrease the expression of LINC00115 in BT20 cells (Fig. 2B). Because upregulation of LINC00115 assciates with TNBC metastasis in our findings, we directly detected its effect on breast cancer cell migration and invasion. Transwell experiments showed that LINC00115 knockdown significantly decreased the migration and invasion of BT20 cells (Fig. 2C,D). These suggest that LINC00115 acts as an oncogene in breast cancer and promotes cell metastasis.

### LINC00115 directly inhibits the expression of miR‐7

**Fig. 3:**
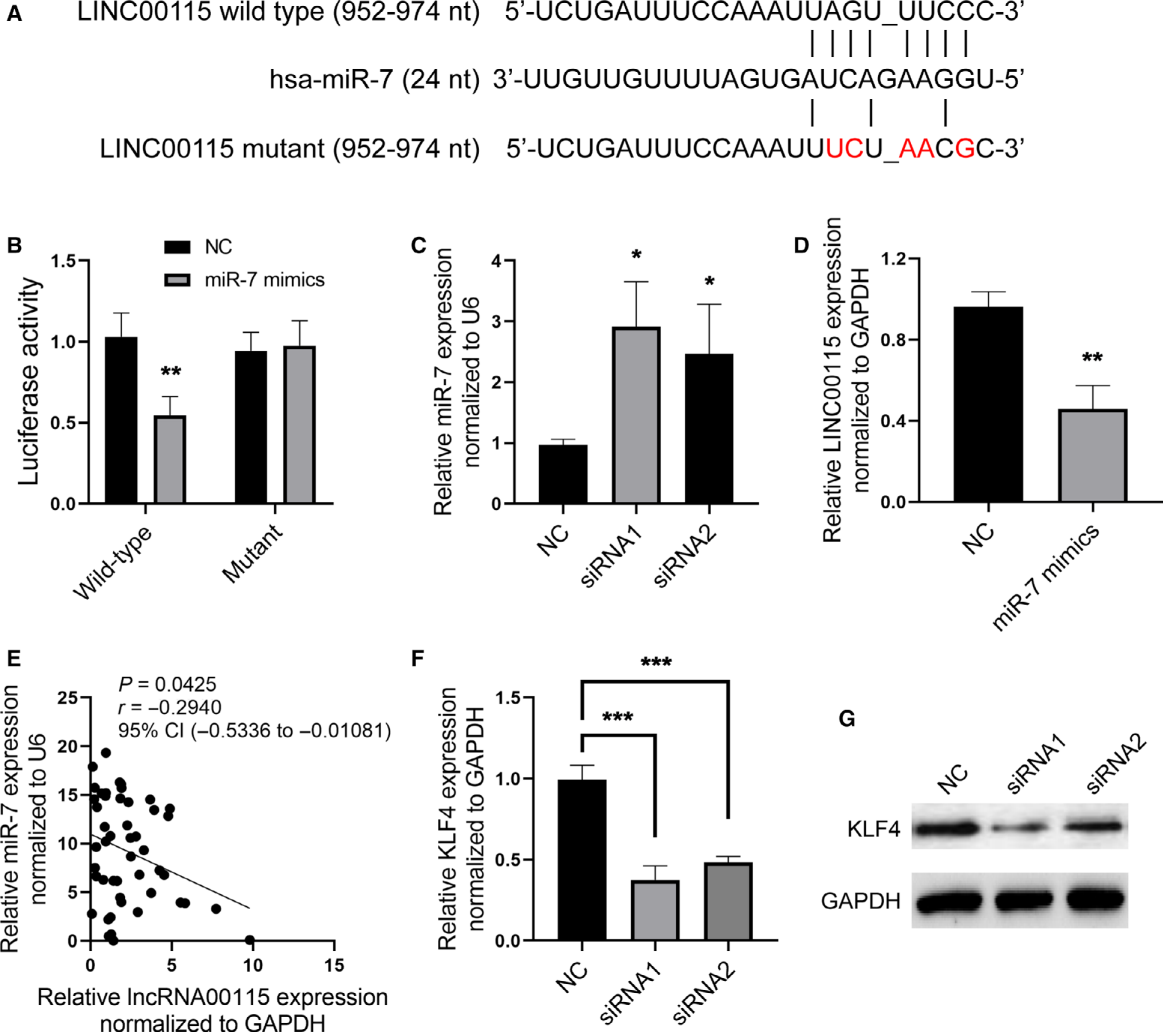
LINC00115 directly inhibits the expression of miR‐7. (A) The binding site of LINC00115 to miR‐7. (B) Bars show the luciferase activity in HEK293 cells which were co‐transfected with miR‐7 and the constructed vectors containing LINC00115 fragment having the binding site of miR‐7, as well as the corresponding control. (C) Bars show the expression of miR‐7 in BT20 cells which were transfected with siRNA1, siRNA2, or NC, respectively. (D) Bars show the expression of LINC00115 in BT20 cells which were transfected with miR‐7 mimics or NC. (E) Pearson correlation analysis shows that there was a negative correlation between the expression of miR‐7 and LINC00115. (F, G) The mRNA and protein expression of KLF4 in BT20 cells which were transfected with NC, siRNA1, or siRNA2. qRT–PCR was used to detect the expression of miR‐7 and LINC00115, and GAPDH served as an internal control for LINC00115 and U6 for miR‐7. The one‐way ANOVA, followed by Tukey’s *post hoc* test, and Student’s *t*‐test were used. Western blotting was used to measure the protein expression of KLF4, and GAPDH served as an internal control. All bars represent data with SD. *P < 0.05; **P < 0.01; and ***P < 0.001.

Next, we ought to explore the underlying mechanism by which LINC00115 promotes breast cancer metastasis. We initially used miRcode (http://mircode.org/) to predict the candidate miRNAs that may bind to LINC00115, and then considered miR‐7 being a potential candidate, because miR‐7 is downregulated in breast cancer and involved in breast cancer metastasis [13]. The binding site of miR‐7 to LINC00115 is shown in Fig. 3A. We then identified the binding site using a luciferase report system. We found that miR‐7 mimics significantly inhibited the luciferase activity in HEK293 cells which had been transfected with the constructed luciferase report vectors containing the wild‐type binding site, but it did not influence the luciferase activity in those with the vectors containing the mutant binding site (Fig. 3B). We further found that LINC00115 knockdown significantly increased the expression of miR‐7, while miR‐7 mimics significantly inhibited the expression of LINC00115 in BT20 cells (Fig. 3C,D). Moreover, we found that there is a negative correlation between the expression of LINC00115 and miR‐7 in TNBC tissues (Fig. 3E). Moreover, we also detected whether LINC00115 can inhibit the expression of KLF4, which is a confirmed target of miR‐7 in breast cancer [14]. The results revealed that LINC00115 knockdown significantly inhibited the mRNA and protein expression of KLF4 (Fig. 3F,G). These suggest that LINC00115 promotes breast cancer through inhibiting miR‐7 expression.

### LINC00115 regulates breast cancer cell migration and invasion via inhibiting miR‐7 *in vitro*

**Fig. 4:**
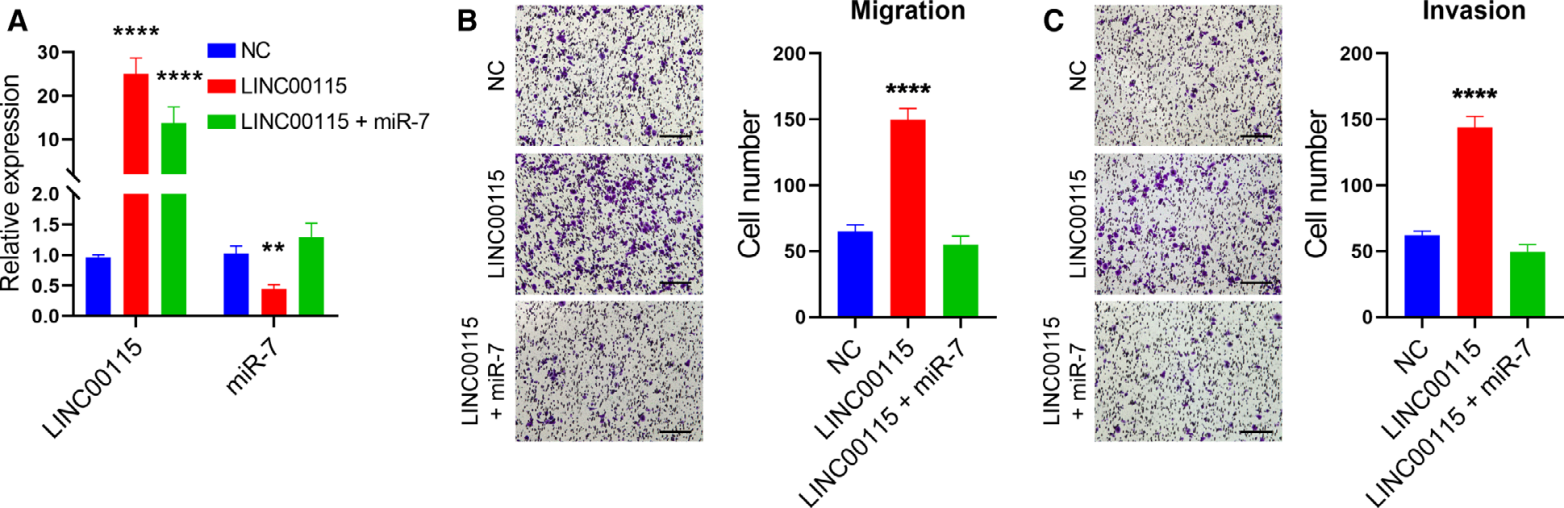
LINC00115 regulates breast cancer cell migration and invasion via inhibiting miR‐7 *in vitro*. (A) Bars show the expression of LINC00115 and miR‐7 in three groups of BT20 cells, including (a) NC; (b) overexpression of LINC00115; and (c) overexpression of both LINC00115 and miR‐7. qRT–PCR was used to detect the expression, and GAPDH served as an internal control for LINC00115 and U6 for miR‐7. Representative micrographs (×100) show the migration (B) and invasion (C) of BT20 cells, respectively, in the three groups. Scale bars represent 25 μm. These experiments were repeated three times. The one‐way ANOVA, followed by Tukey’s *post hoc* test, was used. All bars represent data with SD. **P < 0.01; ****P < 0.0001.

To further determine whether LINC00115 enhances breast cancer cell migration and invasion through regulating miR‐7, LINC00675‐overexpressing vectors and miR‐7 mimics were co‐transfected into BT20 cells, and their expression was measured (Fig. 4A). Transwell assays showed that miR‐7 mimics significantly rescued LINC00115‐induced migration and invasion of BT20 cells (Fig. 4B,C). These suggest that LINC00115 promotes breast cancer cell metastasis through inhibiting miR‐7.

## Discussion

Lymph node‐metastatic TNBC causes markedly poorer outcome [15]. lncRNA expression profiles may serve as potential biomarkers for the diagnosis and prognosis of breast cancer [16, 17]. Lv *et al*. [18] reported four lncRNAs that may serve as new biomarkers to distinguish TNBC from non‐TNBC. Recently, Xu *et al*. [10] used the GEO and TCGA database, and their own high‐throughput sequencing data to find multiple lncRNAs that are overexpressed in breast cancer tissue compared with normal tissue, and some of them are associated with worse survival outcome. More interestingly, LINC00115 does not respond to estrogen, suggesting that this lncRNA may be associated with TNBC. In the present study, we found that LINC00115 expression is increased in TNBC tissue versus matched normal tissue, suggesting that its expression signature may serve as a novel biomarker for differentiating lymph node‐metastatic TNBC from those with nonmetastatic TNBC. However, the number of specimens is modest, and a large‐scale validation needs to be performed in the future.

We selected four breast cancer cell lines (MCF7, SKBR3, BT474, and BT20), among which BT20 is a cell line with negative tests of estrogen, progesterone, and excess HER2, and without BRCA1 mutation. Moreover, we also found that BT20 cells have a higher expression of LINC00115 than the other three cell lines. These suggest that BT20 cell line is suitable for researching the biological function. There are other studies that also used these cell lines to investigate breast cancer metastasis [19, 20].

Next, we used specific siRNAs to inhibit the expression of LINC00115. The lncRNA location in cells can influence the effect of the lncRNA siRNA on inhibiting its expression [21]. Although we are not clear about the location of LINC00115 in breast cancer cells, the specific siRNAs can markedly inhibit the expression of LINC00115 and repress the migration and invasion of BT20 cells. Future studies should identify the exact distribution of LINC00115 in breast cancer cells.

We further investigated the underlying mechanism by which LINC00115 promotes breast cancer cell metastasis. We used miRcode to predict the candidate miRNAs that may bind to LINC00115. This tool has been widely used to predict the interaction between miRNA and lncRNA, as well as miRNA and mRNA [22]. We considered that miR‐7 might be a potential candidate, because miR‐7 has been well known in breast cancer metastasis. For example, miR‐7 inhibits the ability of breast cancer stem‐like cells to spread into the brain through modulating KLF4 [14]; miR‐7 directly inhibits SETDB1 and reverses the epithelial–mesenchymal transition of breast cancer stem‐like cells. Our findings indicate that LINC00115 directly inhibits the expression of miR‐7 in breast cancer cells, while this regulatory mechanism has been also suggested in lung cancer [11]. We further confirmed that LINC00115 promotes breast cancer metastasis via inhibiting miR‐7.

Collectively, our findings indicate that LINC00115 expression is increased in lymph node‐metastatic TNBC tissue compared with nonmetastatic TNBC tissue, as well as matched normal breast cancer tissue; LINC00115 promotes breast cancer cell metastasis by directly inhibiting miR‐7. These suggest that LINC00115 may be a potential therapeutic target for TNBC.

## Materials and methods

### Clinical specimen

A total of 48 patients with TNBC were enrolled in this study. The mean age of the 48 women was 58, and 25 women had lymph node metastasis and the others without any metastasis. Forty‐eight pairs of TNBC tissues and matched normal tissues were collected from the consenting individuals according to the protocols approved by the Ethics Review Board at the Second Affiliated Hospital of Nanchang University from April 2015 to July 2018. The tissues were cut into about 1 × 1 cm^2^ after surgery and stored in nitrogen liquid immediately. The matched normal tissues were collected at a distance of more than 5 cm from the tumor, and all tumors were histologically identified as TNBC. The patients prior to the surgery were not subject to any therapeutics, including chemotherapy or radiotherapy. The experiments were undertaken with the understanding and written consent of each subject. The study methodologies conformed to the standards set by the Declaration of Helsinki.

### Cell culture

Four breast cancer cell lines (MCF7, SKBR3, BT474, and BT20) were purchased from the American Type Culture Collection (ATCC; Manassas, VA, USA). MCF‐7 cells were cultured in RPMI‐1640 (Gibco, Carlsbad, CA, USA) supplemented with 10% FBS (Gibco, Grand Island, NY, USA), and SKBR3, BT474, and BT20 cells were cultured in Dulbecco’s modified Eagle’s medium (Gibco) supplemented with 10% FBS (Gibco). Cells were kept at 37 °C in a humidified incubator with 5% CO_2_.

### Total RNA isolation and quantitative reverse transcription polymerase chain (qRT–PCR)

Total RNAs were extracted from the breast cancer and normal tissues and cells using a TRizol reagent (Invitrogen, Carlsbad, CA, USA) according to the manufacturer’s instruction. qRT–PCR for detecting LINC00115 was performed using a QuantiTect SYBR® Green RT‐PCR Kit (Qiagen, Hilden, Germany) according to the manufacturer’s instruction, and GAPDH was used to normalize the expression of LINC00115 in all samples. The sequences of the primers are shown as follows: 5′‐TGGCTTGTCTTCCATCGTCC‐3′ (forward) and 5′‐ GCACGAGGGTTGTTACAGGA‐3′ (reverse) for LINC00115 and 5′‐ACCACAGTCCATGCCATCAC‐3′ (forward) and 5′‐ TCCACCCTGTTGCTGTA‐3′ (reverse) for GAPDH. For mature miR‐7 expression analysis, the ABI miRNA reverse transcription kit (Applied Biosystems, Foster City, CA, USA) was used along with miR‐7‐specific primers (Applied Biosystems), and then, the qPCR was performed on the ABI 7500 Thermocycler (Applied Biosystems) according to the manufacturer’s protocol. U6 was used to normalize the expression of miR‐7 in all samples.

### Transfection

The specific siRNAs for LINCC00115 were synthesized from Sangon Biotech (Shanghai, China), and miR‐7 mimics were purchased from GenePharma (Shanghai, China). BT20 cells were cultured in six‐well plates up to 70% convergence and transfected with the siRNAs or miR‐7 mimics using Lipofectamine 2000 reagent (Invitrogen) according to the manufacturer’s instruction. The sequences of the siRNAs are shown as follows: siRNA1, 5′‐AAGGATGACCTGGATTGTCCT‐3′; and siRNA2, 5′‐AACACCGACAGTGAAGGAGAG‐3′.

### Luciferase assay

HEK293 cells were cultured in a 24‐well plate and grown up to 70% coverage. The pGL4 Luciferase Reporter Vectors (Promega, San Luis Obispo, CA, USA) containing LINC00115 fragment that has the wild‐type or mutant binding site of miR‐7 were constructed. The wild‐type or mutant vectors combined with the Renilla vectors, as well as miR‐7 mimics or negative control (NC), were transfected into HEK293 cells, respectively. The luciferase assay was performed according to the manufacturer’s protocol. These experiments were repeated three times.

### Migration and invasion assay

Twenty‐four hours after BT20 cells were transfected with the siRNAs or miR‐7 mimics, or co‐transfected with them both, the cells were suspended and diluted into a density of 1 × 10^5^ per mL using the culture medium without FBS, and then, 200 μL of the medium containing the cells was added into the transwell chamber (Millipore, Bedford, MA, USA) with (for invasion) or without (for migration) the Matrigel (BD, Franklin Lakes, NJ, USA). The chamber was placed in a 24‐well plate, where 600‐μL culture medium with FBS (Gibco) was added. After 24 h, the chambers were stained with crystal violet for 10 min, and the cells in the inside of the chambers were wiped off, and then, the migrated cells on the outside of the chambers were calculated under a microscope.

### Western blotting

Total proteins were isolated from BT20 cells using RIPA reagent (Invitrogen). The proteins were subject to separating in a 10% SDS/PAGE gel and then transferred onto a poly(vinylidene difluoride) (PVDF) membrane (Millipore). The PVDF membrane was incubated with a blocking buffer (3% BSA) for 1 h at room temperature and then incubated with anti‐KLF4 [Cell Signaling Technology (CST), Danvers, MA, USA; rabbit mAb #12173; 1 : 1000] or anti‐GAPDH (CST; rabbit mAb #5174; 1 : 1000) primary antibodies overnight at 4 °C. Then, the PVDF membrane was incubated with the secondary antibodies (Zhongshan Biotechnology, Beijing, China; goat anti‐rabbit; 1 : 10 000) for 1 h at room temperature. Finally, the PVDF membrane was exposed by using a SuperSignal West Dura Extended Duration Substrate Kit (Thermo Fisher Scientific, Beijing, China).

### Statistics

All data are presented as the means ± standard deviation (SD). Comparison between two groups was analyzed by Student’s *t*‐test or nonparametric Mann–Whitney–Wilcoxon test. The difference among more than two groups was analyzed by the one‐way ANOVA, followed by Tukey's *post hoc* test. A *P* value < 0.05 was considered statistically significant. All statistical analyses were performed using the graphpad prism 8.0 (GraphPad Software Inc, San Diego, CA, USA) and spss 19.0 software (IBM company, Chicago, IL, USA).

## Conflict of interest

The authors declare no conflict of interest.

## Author contributions

LG designed this study. CY and LG drew the manuscript. CY, XL, and SD performed the experiments and analyzed the data in this study.
